# Successful management of cavernous sinus thrombosis without anticoagulation: a neuro-ophthalmologic case report

**DOI:** 10.3389/fneur.2026.1788368

**Published:** 2026-05-20

**Authors:** Xiao-ou Wang, Cui-zhu Hu, Yi-meng Niu, Wen Tian

**Affiliations:** Department of Geriatric, The First Hospital of China Medical University, Shenyang, China

**Keywords:** antibiotic therapy, anticoagulation, case report, cavernous sinus thrombosis, magnetic resonance imaging, neuro-ophthalmology, septic thrombosis

## Abstract

Cavernous sinus thrombosis (CST) is a rare and life-threatening neuro-ophthalmologic emergency. The role of adjunctive anticoagulation in infection-driven CST—especially when patients respond rapidly to antibiotics—remains a clinically significant dilemma. We report the case of a 50-year-old male presenting with acute right periorbital swelling, proptosis, ocular pain, and headache. Magnetic resonance imaging (MRI) confirmed right cavernous sinus thrombosis secondary to bilateral sinusitis. After multidisciplinary review, anticoagulation was withheld due to the patient’s prompt clinical improvement on targeted intravenous antibiotics (meropenem/ertapenem) and concerns regarding bleeding risk. Complete symptomatic and radiological recovery was achieved, as evidenced by serial MRI follow-up over eight months. This case demonstrates that in selected patients with septic CST and a treatable infectious source, aggressive antibiotic therapy alone may lead to full resolution. It highlights the value of individualized management and contributes to the ongoing discussion on the timing and necessity of anticoagulation in this complex condition.

## 1 Introduction

Cavernous sinus thrombosis (CST) is a rare and severe condition, with an estimated annual incidence ranging from 0.2 to 1.6 cases per 100,000 individuals (1). As a critical neuro-ophthalmologic emergency, it most commonly originates from the spread of infection in the mid-face or paranasal sinuses (2). The classic clinical presentation includes fever, headache, and ocular manifestations—such as periorbital edema, proptosis, and ophthalmoplegia—resulting from the involvement of cranial nerves III, IV, V1, V2, and VI within the cavernous sinus (3). Importantly, proptosis and periorbital edema are not directly caused by cranial nerve involvement, but rather result from impaired venous drainage of the orbit due to obstruction of the cavernous sinus (4). This leads to venous congestion, increased orbital pressure, and reduced venous outflow, which are key contributors to the risk of visual impairment (1, 3). This pathophysiological mechanism also provides a rationale for anticoagulation in selected cases to improve venous hemodynamics (1, 3). Without treatment, CST is associated with significant risks of permanent vision loss, severe neurological deficits, and mortality (5).

Management is centered on urgent infection control and, in many reported cases, systemic anticoagulation to prevent thrombus progression and promote recanalization (3). However, it should be emphasized that much of the evidence supporting anticoagulation is derived from studies on cerebral venous thrombosis (CVT), which encompass heterogeneous etiologies and frequently exclude infection-driven cases (6). As such, the direct applicability of these findings to septic CST remains uncertain (3). Although current CVT guidelines generally recommend early anticoagulation, these recommendations are largely based on non-septic populations and may not fully capture the distinct pathophysiological features of septic CST, where infection, inflammation, and venous obstruction interact in a more complex and dynamic manner (3, 6, 7).

Nevertheless, the use of anticoagulation remains controversial, particularly in septic CST, and lacks universal standardization (8). A central point of debate is whether anticoagulation is necessary in patients who respond rapidly and favorably to antibiotic therapy alone (9). This case report presents the successful management of a septic CST patient without anticoagulation. It serves to contribute evidence to this ongoing clinical discussion and underscores the importance of thorough neuro-ophthalmologic evaluation and imaging in guiding individualized treatment.

## 2 Case presentation

A 50-year-old male presented on October 25, 2021, with a three-day history of progressive right periorbital swelling, proptosis, and deep orbital pain in the absence of discharge, accompanied by right-sided frontal headache.

### 2.1 Clinical and laboratory findings

Vital signs were within normal limits. Neuro-ophthalmologic examination demonstrated marked right periorbital edema and chemosis. Best-corrected visual acuity was 20/20 in both eyes. Pupils were equal and reactive, with no relative afferent pupillary defect. Ocular motility was full bilaterally. Funduscopy revealed sharp optic disc margins without venous engorgement or retinal hemorrhages. Laboratory investigations showed mild leukocytosis (white blood cell count 8.64 × 10^9^/L) with neutrophilia (5.82 × 10^9^/L, 67.4%), along with an elevated plasma D-dimer level (1.11 μg/mL), findings consistent with an active inflammatory response and a prothrombotic state.

Importantly, the patient had no documented history of major bleeding disorders, prior anticoagulant therapy, or underlying hematologic disease. Therefore, the consideration of bleeding risk was precautionary rather than based on a clearly defined high-risk condition. This assessment reflected an individualized clinical judgment, taking into account the potential hemorrhagic risks associated with anticoagulation in the setting of acute infection and systemic inflammation, rather than a pre-existing predisposition to bleeding.

### 2.2 Imaging

Contrast-enhanced magnetic resonance imaging (MRI) of the brain and orbits, performed on October 26, 2021, demonstrated filling defects and abnormal enhancement within the right cavernous sinus, confirming thrombosis. These filling defects represent a key radiological hallmark of intraluminal thrombus formation and provide direct evidence supporting the diagnosis of cavernous sinus thrombosis rather than secondary inflammatory changes. Importantly, these imaging findings should be interpreted as primary radiological indicators of cavernous sinus thrombosis, rather than indirect manifestations of adjacent sinus infection, thereby strengthening the diagnostic specificity of MRI in this context.

Associated findings included dilation of the right superior ophthalmic vein and mild enhancement of the ipsilateral lateral rectus muscle. The dilation of the superior ophthalmic vein further indicates impaired venous outflow from the orbit, reflecting venous congestion secondary to obstruction within the cavernous sinus. Furthermore, the involvement of the superior ophthalmic vein provides complementary hemodynamic evidence of cavernous sinus obstruction, reinforcing the diagnosis of CST by linking structural thrombus formation with its functional consequences on orbital venous drainage.

**Figure 1:**
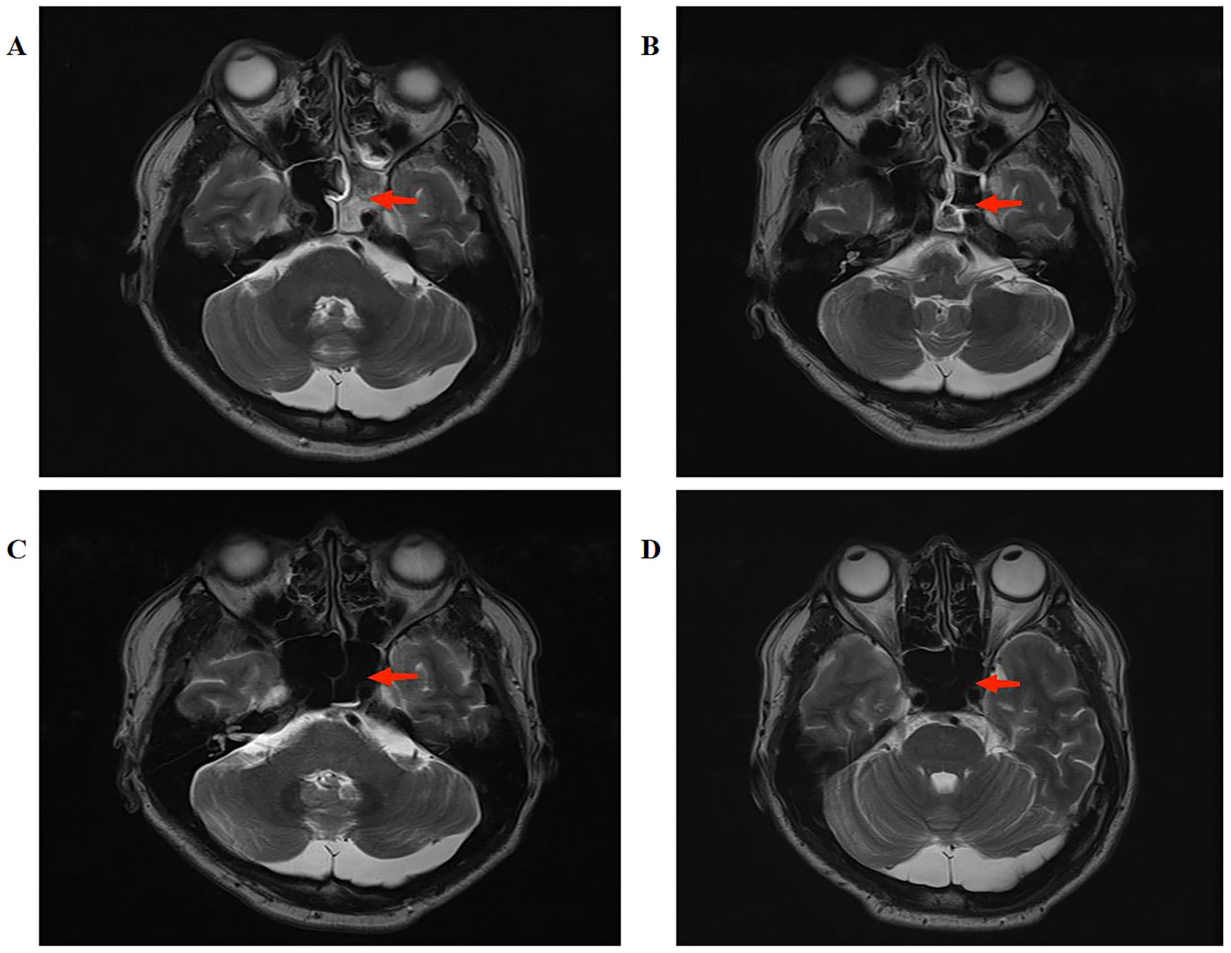
Contrast-enhanced MRI demonstrating cavernous sinus thrombosis at presentation **(A)**; follow-up MRI at 1 month **(B)**; at 3 months **(C)**; and at 8 months **(D)**, showing progressive resolution.

Concurrent bilateral ethmoid and sphenoid sinusitis was noted, indicating the likely septic source (Figure 1A). Together, these imaging features support a pathophysiological process characterized by septic thrombosis with secondary orbital venous drainage impairment, rather than isolated sinusitis. These imaging findings help distinguish CST from isolated sinusitis. They are especially useful for directly visualizing cavernous sinus involvement and associated venous outflow impairment. This also addresses potential diagnostic ambiguity in imaging interpretation. Additional contrast-enhanced MRI findings at presentation and during follow-up are shown in Supplementary Figure 1. These findings further illustrate the dynamic evolution of cavernous sinus abnormalities and their resolution with treatment. Collectively, these findings define a coherent and diagnostically specific imaging profile of septic cavernous sinus thrombosis, enabling clear differentiation from isolated sinusitis or non-thrombotic inflammatory conditions.

### 2.3 Management and clinical course

A diagnosis of septic cavernous sinus thrombosis secondary to sinusitis was made. Empirical intravenous meropenem (2 g every 8 h) was initiated. Notably, anticoagulation was not initiated at the time of admission. This decision was guided by the relatively mild clinical presentation, characterized by preserved visual acuity, absence of cranial nerve deficits, and lack of retinal signs suggestive of significant venous congestion. These initial findings supported a cautious, individualized therapeutic approach rather than immediate implementation of standard combined therapy. Due to clinical improvement and a documented penicillin allergy, therapy was switched to intravenous ertapenem (1 g daily) for a total of six days. A multidisciplinary team, including neurologists, infectious disease specialists, and ophthalmologists, conducted a comprehensive evaluation. In light of the patient’s rapid symptomatic improvement within 48 h following antibiotic initiation, together with a careful consideration of potential bleeding risks, the decision to withhold anticoagulation was reaffirmed through multidisciplinary consensus.

### 2.4 Outcome and follow-up

The patient showed marked clinical improvement within 48 h of initiating antibiotic therapy, with reduction in periorbital swelling and pain. Complete resolution of ocular symptoms and headache occurred progressively over the subsequent several days. Subsequently, inflammatory markers, including C-reactive protein and procalcitonin, as well as D-dimer levels, normalized. A one-month follow-up MRI (Figure 1B) showed significant resolution of the cavernous sinus thrombosis and associated orbital changes. Subsequent MRI scans at three months (Figure 1C) and eight months (Figure 1D) confirmed sustained anatomical resolution. The patient completed a course of oral moxifloxacin upon discharge and remained asymptomatic at final follow-up.

## 3 Discussion

It is essential to distinguish CST from other forms of CVT, as their underlying pathophysiology and therapeutic priorities diverge considerably (3, 7). While routine anticoagulation is broadly supported by the general CVT literature, the evidence base specific to septic CST remains limited and methodologically heterogeneous (3, 6, 10). Consequently, findings from standard CVT guidelines warrant cautious application when managing infection-driven CST (3, 6).

Importantly, septic CST represents a distinct clinicopathological entity characterized by a dynamic interplay between infection, inflammation, and venous obstruction, rather than a purely thrombotic process as commonly observed in non-infectious CVT (3, 10, 11). This distinction has direct therapeutic implications, as the primary treatment priority in septic CST is rapid and effective control of the infectious source, which may secondarily mitigate thrombus propagation (3, 12). In contrast, anticoagulation—while beneficial in many CVT settings—may play a more variable and context-dependent role in septic CST, particularly in patients who exhibit early and sustained clinical improvement following targeted antimicrobial therapy (3, 10).

Furthermore, much of the current evidence supporting anticoagulation originates from heterogeneous CVT populations that often exclude or underrepresent infection-driven cases (7, 10). As such, extrapolation of these findings to septic CST should be undertaken with caution, especially in clinical scenarios where disease severity is limited and the response to antibiotic therapy is rapid (3, 5). In this context, individualized risk–benefit assessment becomes central to therapeutic decision-making, rather than strict adherence to generalized treatment paradigms (3, 5).

This distinction provides the conceptual foundation for the present case discussion, in which a patient with septic CST was successfully treated with antibiotics alone, offering a pragmatic perspective on key diagnostic and therapeutic challenges in neuro-ophthalmology.

### 3.1 Neuro-ophthalmologic presentation and diagnostic paramountcy

The patient presented with acute periorbital edema, proptosis, and pain—a classic, though variable, neuro-ophthalmologic syndrome that should raise immediate suspicion for CST, even in the absence of overt ophthalmoplegia at initial assessment (11). This highlights the importance of maintaining a high index of clinical suspicion, as cranial neuropathies may develop over time. In such cases, neuroimaging is essential for confirmation. Contrast-enhanced MRI with magnetic resonance venography (MRV) remains the diagnostic gold standard, enabling direct visualization of the thrombus, assessment of its extent, and—critically—identification of a potential septic source such as sinusitis (13, 14). In this case, the diagnosis of CST was supported not only by sinus-related findings but also by direct imaging evidence of cavernous sinus involvement and venous outflow disturbance. This convergence of corroborative findings substantially increased diagnostic confidence. In the present case, the rapid identification of concomitant sinusitis was pivotal, as it guided immediate and targeted antimicrobial therapy, the most urgent intervention in septic CST.

### 3.2 Anticoagulation dilemma in septic CST: contextualizing our decision

The decision to withhold anticoagulation represents a central and controversial aspect of this case’s management. Although anticoagulation is strongly recommended for cerebral venous thrombosis to reduce thrombus propagation and recurrence, its role in septic CST is less clearly defined (15). Current guidelines emphasize individualized decision-making, particularly when bleeding risk is significant or when the underlying pathophysiology is primarily infectious (16).

In this context, early clinical indicators played a pivotal role in guiding therapeutic strategy. The preservation of visual function, together with the absence of overt neurological or cranial nerve deficits at presentation, suggested a relatively limited extent of neuro-ophthalmologic involvement. These findings, when considered alongside the infection-driven pathophysiology of septic CST, supported a cautious and individualized initial management approach rather than the immediate initiation of anticoagulation therapy (3, 15).

Furthermore, it is important to recognize that a substantial proportion of the existing evidence base is derived from studies evaluating advanced interventions, such as endovascular therapy, which typically involve critically ill patients with severe or refractory disease (17). These populations differ markedly from patients with early-stage or infection-responsive CST, as presented in this case. Consequently, extrapolation of findings from such high-risk cohorts to milder clinical scenarios should be approached with caution, particularly when the disease course is rapidly reversible with targeted antimicrobial therapy.

In the present case, the relatively mild clinical presentation—reflected by preserved visual acuity, absence of cranial nerve deficits, and lack of retinal venous congestion—likely represents an earlier or less extensive stage of disease involvement. This clinical profile may have contributed to the rapid and favorable response to antibiotic therapy alone. In contrast, patients with more advanced or extensive CST may experience a more aggressive disease course and could require earlier initiation of anticoagulation or other intensified therapeutic strategies. These observations underscore the importance of stratifying treatment decisions according to disease severity and clinical trajectory, rather than applying uniform management approaches across heterogeneous patient populations (15).

Several case-specific factors supported a conservative approach (Table 1). First, the infectious source was promptly identified and aggressively treated with broad-spectrum antibiotics, to which the patient demonstrated a rapid clinical response within 48 h. This aligns with the pathophysiological understanding that controlling the infectious nidus is the primary driver of recovery in septic CST (18). Second, serial imaging provided objective evidence of thrombus resolution concurrent with antibiotic therapy, suggesting effective removal of the pro-thrombotic inflammatory stimulus (5, 19). Finally, a multidisciplinary team carefully evaluated the patient’s individual bleeding risk (19).

**Table 1:** Key clinical factors influencing the decision to withhold anticoagulation in septic CST.

Domain	Findings in this case	Clinical implication
Etiology	Confirmed septic CST secondary to sinusitis	Infection control prioritized
Response to antibiotics	Marked improvement within 48 h	Suggests inflammation-driven thrombosis
Imaging evolution	Progressive thrombus resolution on serial MRI	Reduced need for anticoagulation
Bleeding risk	Considered non-negligible	Favored conservative strategy
Multidisciplinary input	Neurology, ophthalmology, infectious disease	Individualized decision endorsed

CST, cavernous sinus thrombosis; MRI, magnetic resonance imaging.

Taken together, these considerations underscore that the role of anticoagulation in septic CST remains incompletely defined (3). In current clinical practice, therapeutic decisions are therefore guided less by universally standardized protocols and more by individualized risk–benefit assessment, taking into account disease severity, response to antimicrobial therapy, and patient-specific risk profiles (3). In this context, individualized decision-making frameworks are particularly critical in septic CST, as both the extent of disease involvement and the trajectory of response to infection-directed therapy can dynamically influence the balance between potential therapeutic benefit and hemorrhagic risk associated with anticoagulation (5).

This case thus illustrates a scenario in which, following nuanced risk–benefit analysis, the potential risks of anticoagulation may outweigh its benefits, reinforcing the value of a tailored rather than universal treatment protocol.

### 3.3 Limitations

The findings from this single case report must be interpreted with caution. Its primary limitation lies in inherent lack of generalizability, as a single observation cannot establish definitive conclusions regarding the safety or efficacy of omitting anticoagulation in broader CST populations. Moreover, the relatively mild disease severity observed in this case—characterized by preserved visual function and absence of cranial nerve involvement—may further restrict the applicability of these findings to patients with more advanced or complicated CST, in whom the therapeutic role and potential benefits of anticoagulation may differ substantially. In addition, the retrospective nature of this report and the absence of a comparator group receiving anticoagulation preclude any direct assessment of relative treatment efficacy. Although advanced vascular imaging modalities such as MRV or computed tomography venography were not performed, the diagnosis was supported by characteristic contrast-enhanced MRI findings and consistent longitudinal imaging changes. Furthermore, although anatomical resolution was thoroughly documented with imaging, formal quantitative assessment of long-term neuro-ophthalmologic function—such as detailed visual fields or diplopia scoring—was not performed. Collectively, these limitations highlight the need for well-designed prospective studies, including multicenter cohorts or registry-based analyses, to better delineate patient subgroups—particularly those with septic etiology and early responsiveness to antibiotic therapy—in whom a non-anticoagulation strategy may be both safe and effective.

## 4 Conclusion

This case report illustrates that septic CST can achieve complete clinical and radiological resolution with timely and targeted antibiotic therapy alone, without adjunctive anticoagulation. It highlights the essential role of prompt MRI-based diagnosis, identification and management of the infectious source, and individualized therapeutic decision-making within a multidisciplinary team. While anticoagulation remains a cornerstone of treatment for most CST patients, this case contributes to emerging evidence that in a carefully selected subset—characterized by a clear infectious etiology, rapid clinical response to antibiotics, and a favorable risk–benefit assessment—a strategy omitting anticoagulation may be a viable and safe alternative. This experience underscores the need for further research to establish refined patient selection criteria for anticoagulation in this complex and vision-threatening condition.

## Data Availability

The original contributions presented in the study are included in the article/Supplementary material, further inquiries can be directed to the corresponding author/s.
