# Supplementary material for: Successful management of cavernous sinus thrombosis without anticoagulation: a neuro-ophthalmologic case report
Source: Front Neurol. 2026 May 20;17:1788368. doi: 10.3389/fneur.2026.1788368 (PMC13229824; doi:10.3389/fneur.2026.1788368)
Supplement: Supplementary file 1 [file Data_Sheet_1.pdf]

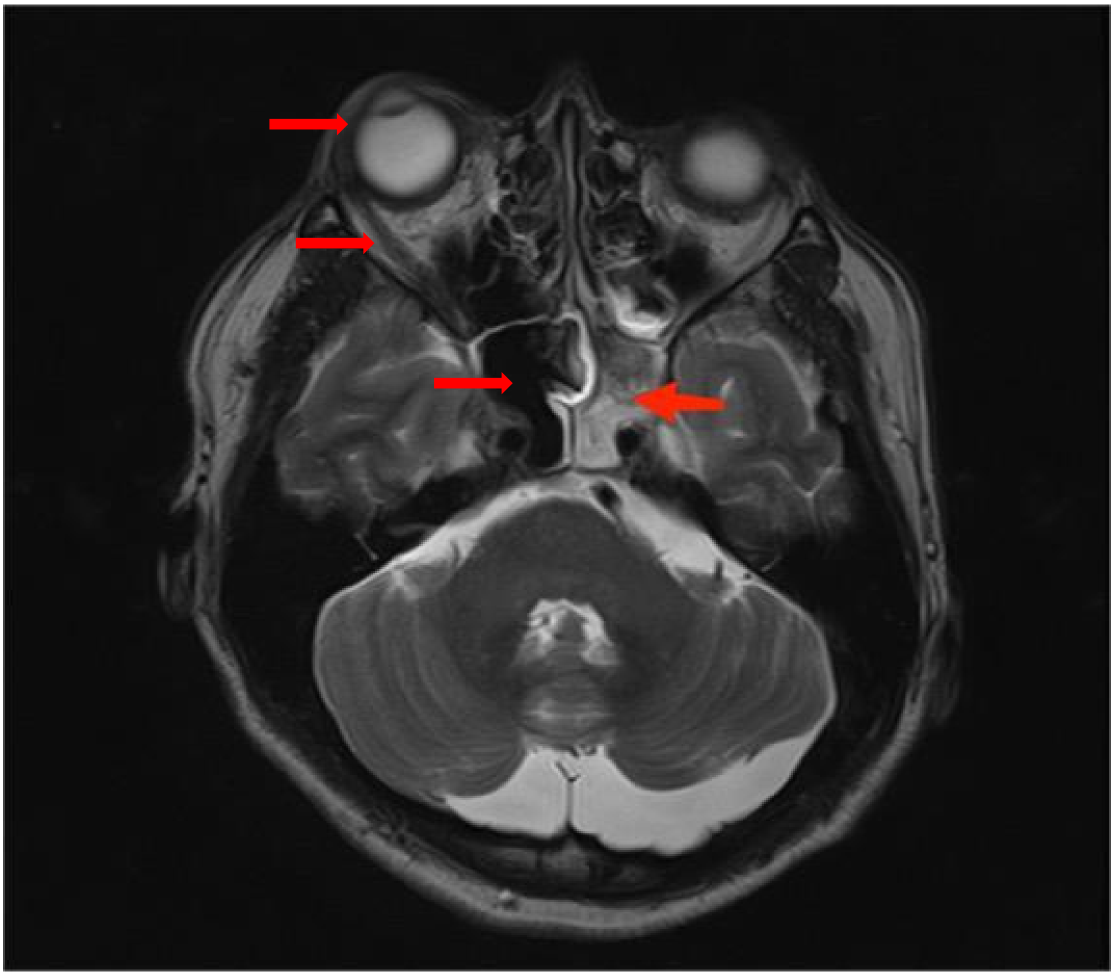

Supplementary Figure 1A. Contrast-enhanced brain MRI of right cavernous sinus thrombophlebitis at presentation.

Notes:

Left upper arrow: Right-sided proptosis.

Left middle arrow: Enlargement of the muscle belly of the lateral rectus muscle.

Left lower arrow: Dilatation of the cavernous sinus, with patchy areas of hyperintense signal on T2-weighted imaging observed within it.

Right arrow: Over-pneumatization of the left sphenoid sinus; the sinus is entirely filled with material that exhibits slightly hyperintense signal on both T1-weighted and T2-weighted imaging.

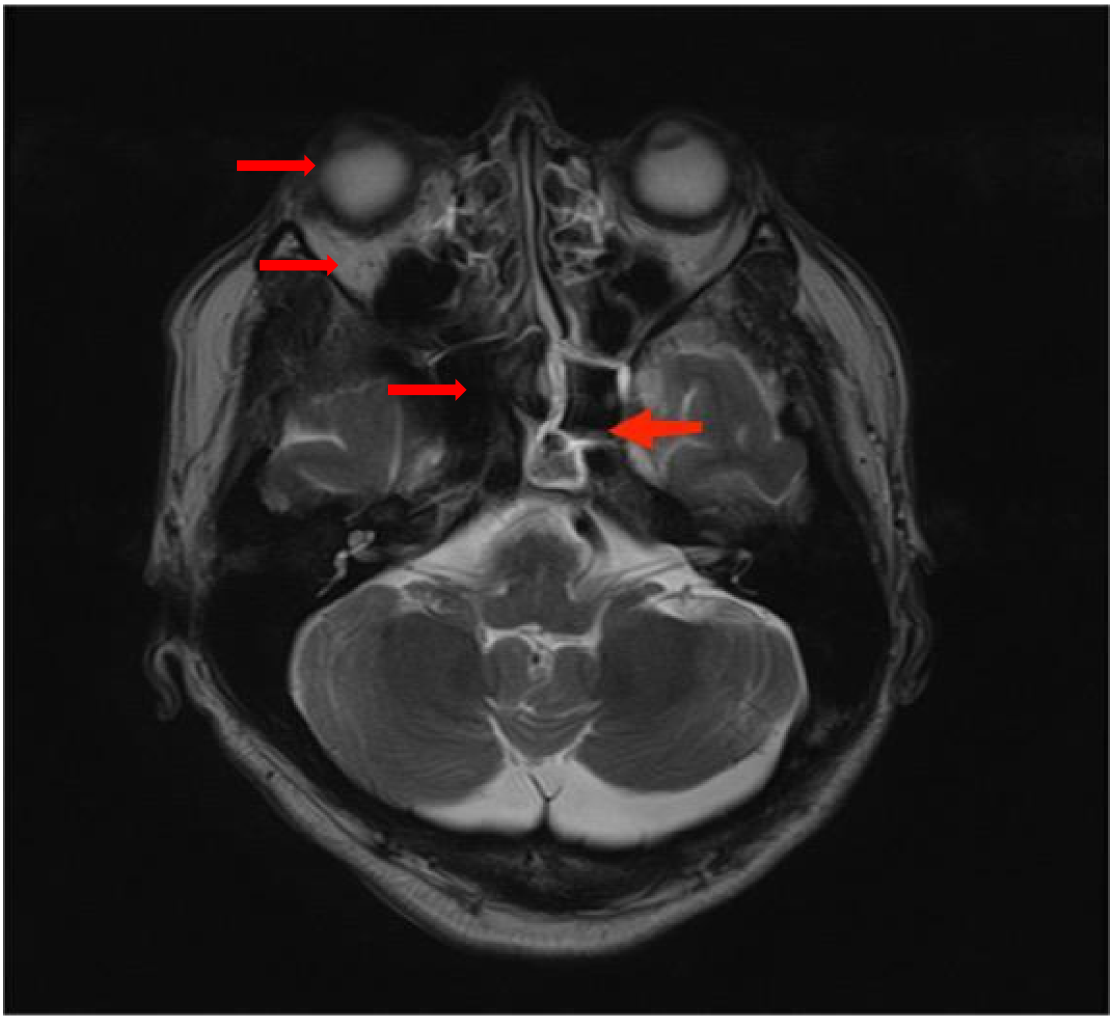

Supplementary Figure 1B. Contrast-enhanced brain MRI of right cavernous sinus thrombophlebitis after 2 weeks of treatment.

Notes:
Left upper arrow: Mild right-sided proptosis.
Left middle arrow: Slight thickening of the muscle belly of the lateral rectus muscle.
Left lower arrow: The cavernous sinus is not enlarged, and no abnormal enhancement is observed.
Right arrow: Mucosal thickening of the left sphenoid sinus; the sinus is completely filled with material demonstrating slightly hypointense signal on T1-weighted imaging and slightly hyperintense signal on T2-weighted imaging, with mucosal enhancement.

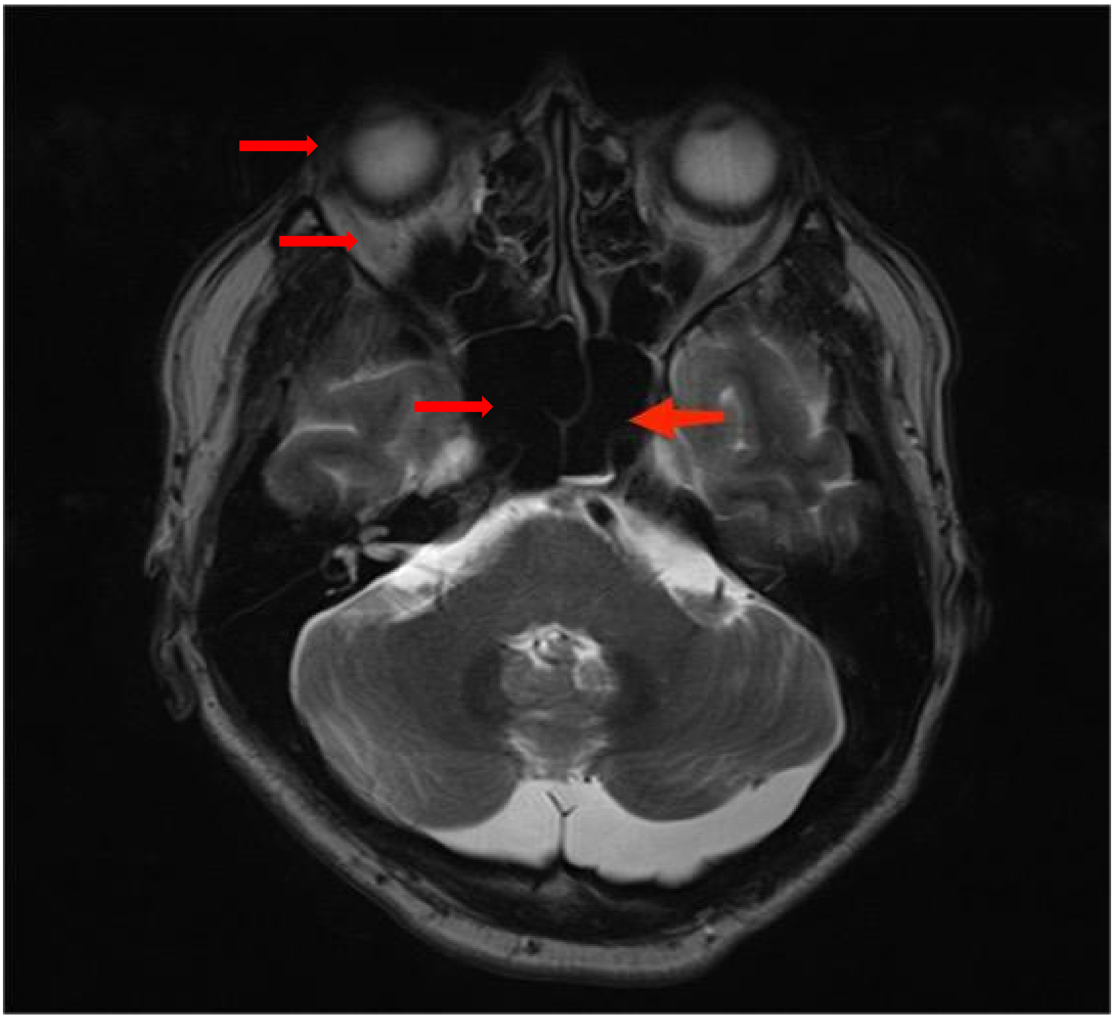

Supplementary Figure 1C. Contrast-enhanced brain MRI of right cavernous sinus thrombophlebitis at the 1-month follow-up after discharge.

Notes:
Left upper arrow: Mild right-sided proptosis.
Left middle arrow: Slight thickening of the muscle belly of the lateral rectus muscle.
Left lower arrow: The cavernous sinus is not enlarged, and no abnormal enhancement is observed.
Right arrow: Slight mucosal thickening of the left sphenoid sinus.

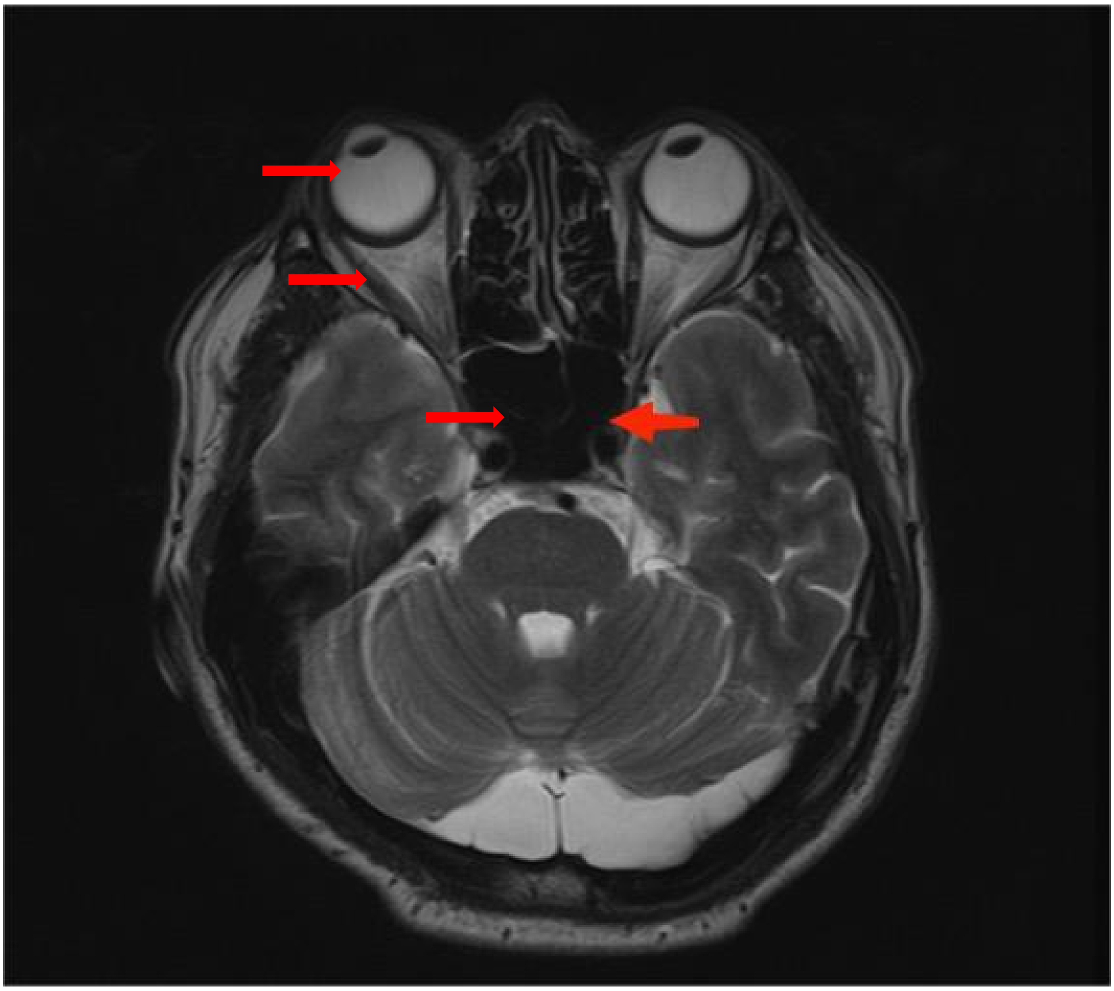

Supplementary Figure 1D. Contrast-enhanced brain MRI of right cavernous sinus thrombophlebitis at the 7-month follow-up after discharge.

Notes:
Left upper arrow: The right eyeball has returned to its normal position.
Left middle arrow: No thickening of the muscle belly of the lateral rectus muscle is observed.
Left lower arrow: The cavernous sinus is not enlarged, and no abnormal enhancement is seen.
Right arrow: No abnormalities are observed in the left sphenoid sinus.
